# Replication slippage of the thermophilic DNA polymerases B and D from the Euryarchaeota *Pyrococcus abyssi*

**DOI:** 10.3389/fmicb.2014.00403

**Published:** 2014-08-07

**Authors:** Melissa Castillo-Lizardo, Ghislaine Henneke, Enrique Viguera

**Affiliations:** ^1^Departamento de Biología Celular, Genética y Fisiología, Facultad de Ciencias, Universidad de MalagaMálaga, Spain; ^2^Laboratoire de Microbiologie des Environnements Extrêmes, UMR 6197, Institut Français de Recherche pour l’Exploitation de la Mer, Université de Bretagne OccidentalePlouzané, France; ^3^CNRS, UMR 6197, Laboratoire de Microbiologie des Environnements ExtrêmesPlouzané, France

**Keywords:** slippage, primer-template misalignment, DNA polymerases, strand displacement activity, Archaea

## Abstract

Replication slippage or slipped-strand mispairing involves the misalignment of DNA strands during the replication of repeated DNA sequences, and can lead to genetic rearrangements such as microsatellite instability. Here, we show that PolB and PolD replicative DNA polymerases from the archaeal model *Pyrococcus abyssi* (*Pab*) slip *in vitro* during replication of a single-stranded DNA template carrying a hairpin structure and short direct repeats. We find that this occurs in both their wild-type (exo+) and exonuclease deficient (exo-) forms. The slippage behavior of *Pab*PolB and *Pab*PolD, probably due to limited strand displacement activity, resembles that observed for the high fidelity *P. furiosus* (*Pfu*) DNA polymerase. The presence of *Pab*PCNA inhibited *Pab*PolB and *Pab*PolD slippage. We propose a model whereby *Pab*PCNA stimulates strand displacement activity and polymerase progression through the hairpin, thus permitting the error-free replication of repetitive sequences.

## INTRODUCTION

Low complexity DNA sequences such as microsatellites (1–9 nt repeat length), including mono, di, and trinucleotide repeats, and minisatellites (unit ≥10 nt) are frequently associated with mutagenesis “hot-spots” in both eukaryotic and prokaryotic genomes (Bierne et al., 1991; Michel, 2000; Aguilera and Gomez-Gonzalez, 2008). These types of sequences are characterized by high instability, consisting of the addition or deletion of repeated units, leading to variations in repeat copy number. Such genetic variations have been termed “dynamic mutations” (Richards and Sutherland, 1992; Pearson et al., 2005). Arrest of the replication machinery within a repeated region is associated with such instability, where primer and template become misaligned (reviewed in Michel, 2000). This process, known as replication slippage, is involved in the generation of deletions or insertions within repeat regions (Viguera et al., 2001a; Lovett, 2004).

**FIGURE 1:**
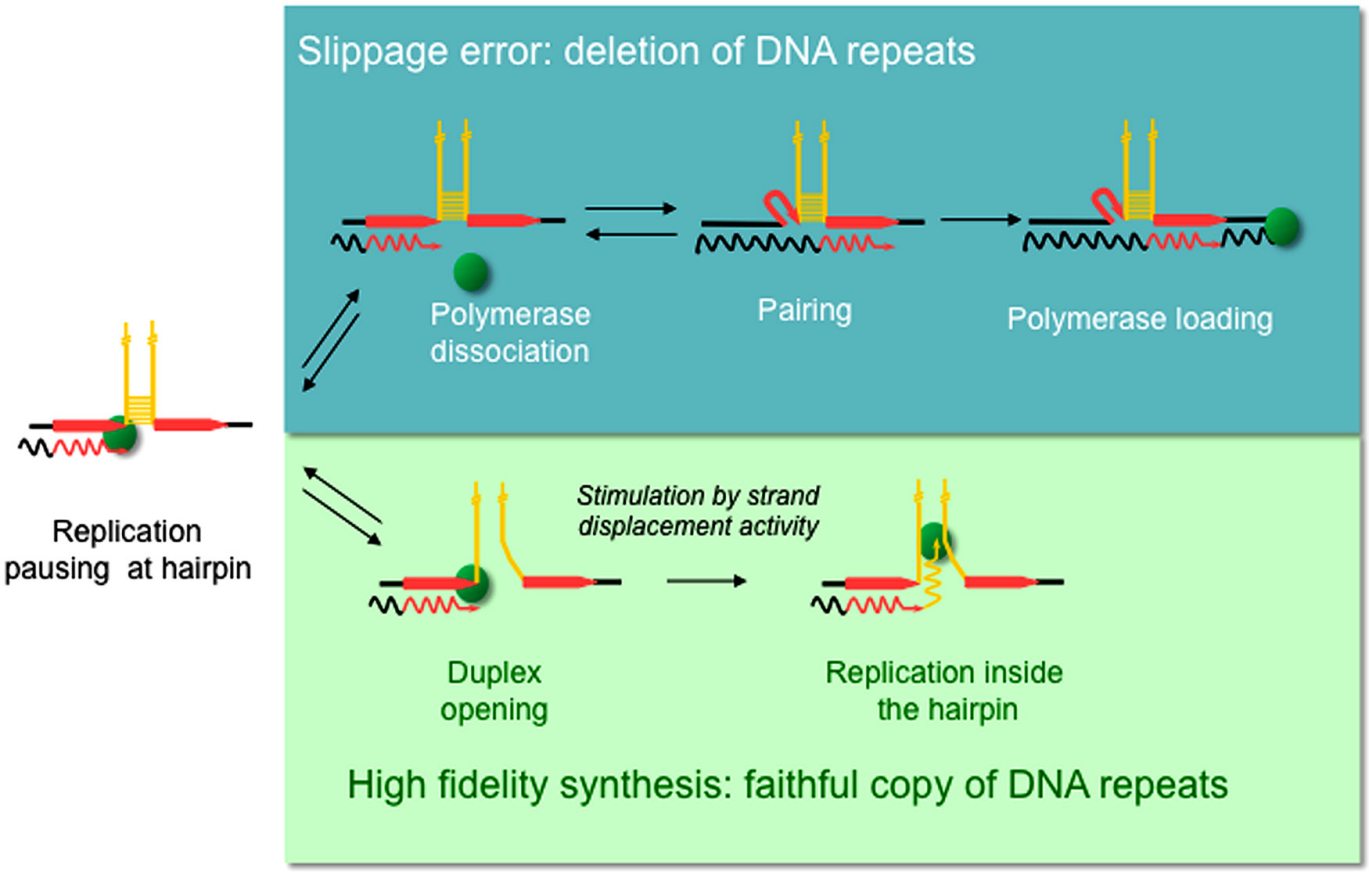
**Model proposed for replication slippage between direct repeats promoted by hairpin-containing templates.** Diagram shows part of a single stranded template (straight lines) with newly synthesized DNA (wavy lines) and the DNA polymerase (green sphere). Direct repeats (red arrows) flank inverted repeats (yellow) that anneal to form a hairpin structure. Slippage-mediated deletion is proposed to involve three sieps (Top): (1) the polymerase pauses within the first direct repeat at the base of the hairpin, (2) polymerase dissociation, and (3) strand misalignment, where the 3’ end of the nascent strand unpairs from the complementary strand and reanneals with the second direct repeat, thus generating a deletion (Viguera et al., 2001a). However, a polymerase with high strand displacement activity is able to open the hairpin duplex (bottom) and replicate the repeat-containing template faithfully.

Replication slippage has been proposed to occur within homopolymeric runs (Kroutil et al., 1996) as well as in short and long tandem repeat sequences (Trinh and Sinden, 1993; Madsen et al., 1993; Tran et al., 1995; Bierne et al., 1996; Feschenko and Lovett, 1998). Repeated DNA sequences are generally characterized by the formation of non-B DNA structures, the majority of which can form intra-strand hairpin loops (Samadashwily et al., 1997; McMurray, 1999; Mirkin, 2007; Sinden et al., 2007). A direct role for replication slippage in the deletion of repeated sequences within hairpin structures has been demonstrated *in vitro* and *in vivo* (d’Alencon et al., 1994; Canceill and Ehrlich, 1996). Slippage-mediated deletions are believed to occur via a three step mechanism as illustrated in **Figure 1** (Viguera et al., 2001a). In this model, the polymerase pauses as it reaches the base of the hairpin after copying the first direct repeat (DR), followed by polymerase dissociation. The 3′ end of the nascent strand then unpairs from the template before reannealing to the second DR. This new primer/template complex is recognized by the polymerase, allowing replication to continue but also generating a deletion.

Several DNA polymerases have been tested for their propensity to slip *in vitro* when replicating hairpin-containing templates. Surprisingly, the replicative DNA polymerase Pol III holoenzyme (HE) from *Escherichia coli* can slip *in vitro* (Canceill and Ehrlich, 1996; Canceill et al., 1999). This is of utmost importance because high fidelity replication is required to maintain genome integrity. Studies on DNA polymerases involved in DNA repair such as *E. coli* Pol I, *E. coli* Pol II, and the T4, T7, and ϕ29 phage DNA polymerases revealed that the strand displacement activity of a DNA polymerase is inversely related to their propensity to slip. DNA polymerases with high strand displacement activity such as ϕ29 or T7 pol exo- (Sequenase^TM^) can progress through template hairpin structures and consequently do not slip. On the other hand, DNA polymerases devoid of strand displacement activity such as *E. coli* DNA Pol II or T4 are blocked at the base of the hairpin, promoting DNA repeat misalignment and subsequent loss of repeat sequences. Depending on the template and the strand displacement activity of a DNA polymerase it is possible for the deletion error rate to exceed the base substitution rate (Kunkel and Bebenek, 2000). In the context of the model proposed for slippage-mediated deletions (**Figure 1**), a polymerase with high strand displacement activity would be able to open the hairpin duplex, avoiding the polymerase dissociation and nascent strand reannealing steps, and thus replicate the repeat-containing template faithfully.

Several thermostable DNA polymerases utilized for PCR can also slip even under the high temperatures used during PCR amplification (Viguera et al., 2001b); **Table 1**. DNA polymerases with high fidelity in terms of base substitution rate, such as *P. furiosus* (*Pfu*Pol), consistently undergo slippage and introduce deletions while replicating hairpin-containing templates (Viguera et al., 2001b). In contrast, a low fidelity DNA polymerase such as *Thermus aquaticus* (*Taq*Pol) can replicate the same hairpin sequence without introducing deletions, although this is dependent on the magnesium concentration used. Other thermostable DNA polymerases endowed with a high strand displacement activity such as *Thermococcus fumicolans* (*Tfu*Pol) or *Bacillus stearothermophilus* (*Bst*Pol) also do not slip when replicating hairpin-containing sequences (Viguera et al., 2001b).

**Table 1:** Effect of magnesium concentration on the slippage of *Pfu*, *Taq*, Vent (50°C), Vent (65°C), *Tfu* and *Bst* polymerases determined previously (Viguera et al., 2001b) and *Pab* PolB, *Pab* PolB exo-, *Pab* PolD, and *Pab* PolD exo- determined in this work.

Polymerase	Magnesium concentration (mM)							
	0.5	1	2.5	5	7.5	10	15	20
*Pfu Pol*	S	S	S	S	S	–	–	*nd*
*Pfu Pol* (native)	–	P/S	P/S	S	S	–	–	*–*
*Taq* Pol	P	P	P	P/S	P/S	S	S	*–*
Vent Pol (50°C)	–	–	S	S	S	S	–	*nd*
Vent Pol (65°C)	–	–	*rcr*	*rcr*	P/S	P/S	–	*nd*
*Tfu* Pol	*nd*	*rcr*	*nd*	*nd*	*nd*	*nd*	*nd*	*nd*
*Bst* Pol	*–*	–	*rcr*	*rcr*	*rcr*	*rcr*	**nd**	*nd*
*Pab* PolB	*–*	P/S	P/S	P/S	P/S	S	S	*–*
*Pab* PolB exo-	*–*	P/S	P/S	P/S	S	S	–	*–*
*Pab* PolD	*–*	S	P/S	P/S	S	S	S	*–*
*Pab* PolD exo-	*–*	P/S	P/S	P/S	S	S	S	S

*The main product obtained for each reaction is shown. S indicates slipped molecules, generated by replication slippage error. P indicates parental molecules, indicative of faithful replication. rcr indicates high molecular weight molecules generated by rolling circle replication as a consequence of the strand displacement activity of a DNA polymerase. nd, not determined.*

We have studied here the biochemical properties of DNA polymerases PolB (*Pab*PolB) and PolD (*Pab*PolD) from the hyperthermophilic euryarchaeon *P. abyssi* in terms of slippage during *in vitro* primer extension reactions. Archaeal replication proteins are more closely related to their eukaryotic than their bacterial equivalents. Euryarchaeal members contain DNA polymerases that belong to both the ubiquitous B family as well as the D family, which is unique to archaea (Ishino et al., 1998; Barry and Bell, 2006; Raymann et al., 2014). Both PolB and PolD have associated 3′-5′ exonuclease activity and moderate strand displacement activity, although PolB cannot displace a RNA-DNA hybrid (Henneke, 2012). However, in the presence of *Pab* Proliferating cell nuclear antigen (PCNA), both *Pab* polymerases show strand displacement activity (Henneke et al., 2005; Rouillon et al., 2007). Moreover, *Pab*PCNA can be loaded onto DNA in the absence of the clamp-loader replication factor C (RF-C), although the presence of this factor does enhance its loading (Rouillon et al., 2007).

In this work, we report that both *P. abyssi* DNA polymerases slip *in vitro* on a template that consists of single-stranded DNA (ssDNA) with a hairpin structure flanked by short direct repeats. In addition, we find that *Pab*PCNA increases replication fidelity of this template by triggering the strand displacement activity of *Pab*polB. Furthermore, we describe the effect of magnesium concentration on the replication slippage of both *Pab* DNA polymerases. These results help toward understanding the dynamics of replication through common non-B DNA structures and identifying the key DNA polymerases involved in replication slippage; a crucial step for understanding genome stability in these organisms.

## MATERIALS AND METHODS

### PROTEINS

*Pab*PCNA,* Pab*pol D, and exonuclease-deficient *Pab*pol D were obtained from G. Henneke (Ifremer, Brest, France). They were cloned, expressed and purified as described (Gueguen et al., 2001; Henneke et al., 2002, 2005; Palud et al., 2008). *Pab*polB (Isis^TM^) and *Pab*polB exonuclease-deficient (*Pyra^TM^* exo-) were purchased from MP Biomedicals. One unit of *Pab* pols corresponds to the incorporation of 1 nmol of total dTMP into acid precipitable material per minute at 65°C in a standard assay containing 0.5 μg (nucleotides) of poly(dA)/oligo(dT)_10:1_ M13 gene protein II (gp II) was purified to homogeneity as described (Greenstein and Horiuchi, 1987). *Thermus thermophilus* SSB was a kind gift from Drs. C. Perales and J. Berenguer (CBM-SO, Madrid). Native *Pfu* Pol was from Stratagene. *Taq* Pol was from Roche Molecular Biochemicals.

### ssDNA TEMPLATE

Construction of the pHP727FXc plasmid has been described previously (Canceill and Ehrlich, 1996). Preparation of ssDNA templates was carried out essentially as described (Canceill and Ehrlich, 1996) with the following modifications: plasmid DNA was extracted using a Maxi Plasmid Kit (Qiagen). Briefly, a specific nick was introduced into the f1 replication origin (+) strand of purified FXc plasmid DNA using the M13 gpII protein. The reaction was stopped with 20 mM EDTA and the products treated with 200 μg/ml proteinase K for 10 min at 55°C, phenol extracted and dialyzed against TE buffer. Nicked strands were removed by exonuclease III digestion (10–40 units per μg of DNA for 1 h at 37°C). Finally, Exo III, nucleotides and oligonucleotides were removed using QIAquick^®;^ PCR (Qiagen) purification kits.

### PRIMER EXTENSION REACTIONS

*Pyrococcus abyssi* pols were tested in a primer extension reaction performed as described (Canceill and Ehrlich, 1996; Canceill et al., 1999). Briefly, 24.3 fmol of a 5′-end fluorescein labeled primer (Applied Biosystems) designated 1233 (5′AGC GGA TAA CAA TTT CAC ACA GGA 3′), were annealed 1235 bases upstream of the palindrome. All 10 μl primer extension reactions contained 25 ng (12.2 fmol) of primed ssDNA, and *Pab* pols that were added to the reaction mixture as indicated in the figure legends. Additionally, reactions contained unless otherwise mentioned 50 mM Tris-HCl (pH 8.8), 50 mM KCl, 10 mM DTT, 2 mM MgCl_2,_ and 200 μM dNTPs. Reactions were performed at 60°C for 30 min, synthesis was arrested by the addition of 25 mM EDTA and 500 μg/ml proteinase K, and the mixture was further incubated for 15 min at 55°C. Reaction products were analyzed by electrophoresis using 0.8% agarose gels under native conditions, run in TBE buffer (89 mM Tris-borate, 2 mM EDTA, pH 8.3) at 2 V/cm for 16 h and visualized with a Typhoon 9400 Variable Mode Imager (Amersham Biosciences, GE Healthcare). Analysis of the results was performed using Image Quant 5.2 software. Quantification analysis was performed with Visilog 6.3 (Visualization Sciences Group. Noesis). A common fixed area was selected at the center of the bands corresponding to parental and heteroduplex molecules. The average gray value of all pixels of each area was obtained and the proportion of parental/heteroduplex was calculated.

*Pfu* Pol and *Taq* Pol were tested as above except that 200 μM dGTP, dATP, and dTTP (each), 40 μM dCTP and 50 μM (2.5 μCi) (α-^32^P)dCTP was used. The reaction buffers were prepared magnesium free as those furnished by the suppliers and contained, in addition to 30 mM NaCl brought by the primed ssDNA, the following ingredients: (i) for *Taq* Pol: 10 mM Tris-HCl pH 8.3, 50 mM KCl; (ii) for Native *Pfu* Pol: 20 mM Tris-HCl pH 8.0, 10 mM KCl, 6 mM (NH_4_)_2_SO_4_, 0.1% Triton^®;^ X-100, 10 μg/ml BSA. After gel electrophoresis, DNA was visualized by direct exposure of the dried gels to Imaging Plates (IP BAS-MP 2040S) and analyzed on a Fujifilm-BAS 1500.

## RESULTS

### EXPERIMENTAL SYSTEM

**FIGURE 2:**
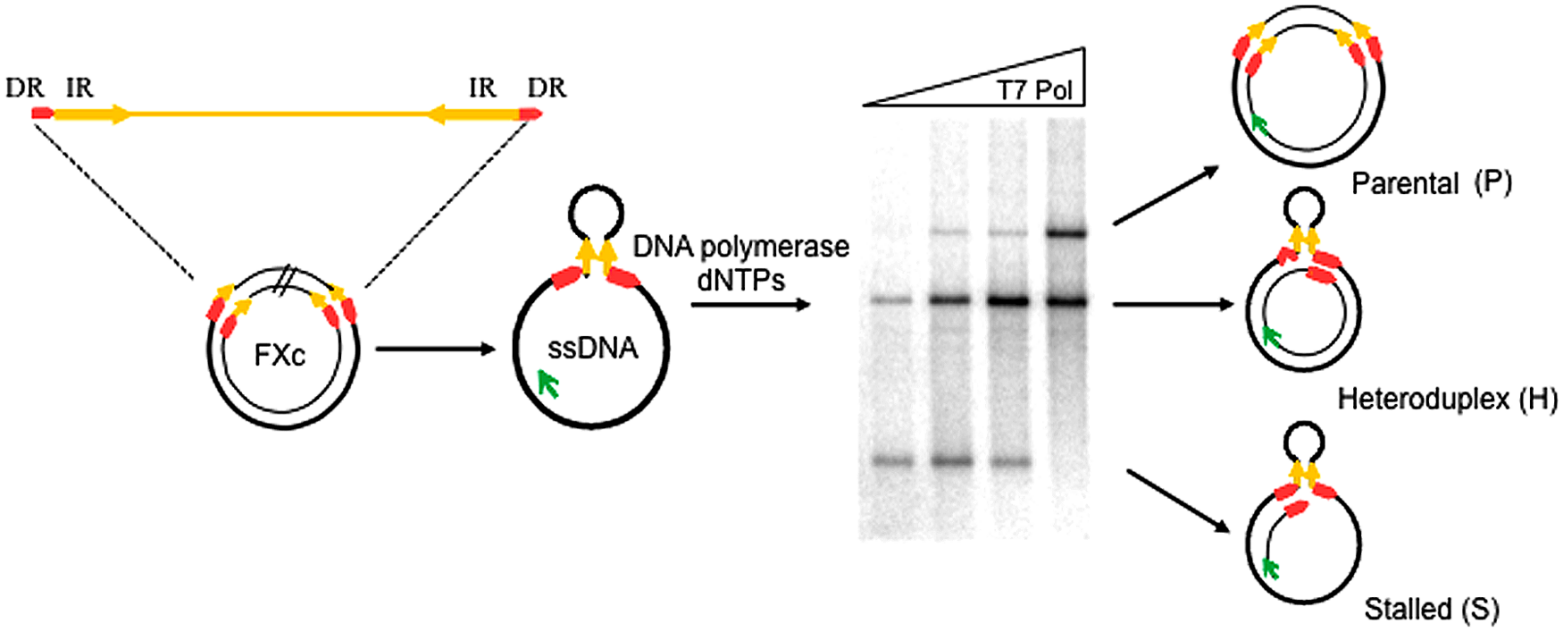
**Experimental assay for the detection of replication slippage.** Schematic representation of the ssDNA template and the different replication products expected after a primer extension reaction, FXc represents the double-stranded pHP727FXc plasmid containing a central 1370 bp region (insert) flanked by 300-bp inverted repeats (IR: yellow arrows) and 27-bp direct repeats (DR; red arrows). ssDNA FXc template is prepared *in vitro* and primer extension reactions performed at 60°C in the presence of a fluorescein-labeled primer (green arrow) and DNA polymerase as described in the “Materials and Methods.” Reaction products are then separated by agarose gel electrophoresis. An assay testing different T7 DNA Pol concentrations is shown in the example. P indicates fully replicated parental molecules. H represents heteroduplex molecules generated after slippage, with one strand lacking one direct repeat unit and the hairpin. S indicates stalled molecules generated by arrest of the polymerase at the base of the hairpin. Bands migrating between S and H correspond to DNA polymerase arrest inside the hairpin. Bands migrating above P corresponds to high molecular weight molecules generated by displacement of the extended primer (Viguera et al., 2001b).

To study whether *P. abyssi* thermostable DNA polymerases (*Pab* pols) promote replication slippage, we performed primer-extension assays using the circular ssDNA template, FXc (Canceill and Ehrlich, 1996; Canceill et al., 1999; Viguera et al., 2001a,b). This template contains two 27 bp direct repeats (DRs) that flank a pair of 300 bp inverted repeats (IR) separated by a 1.3 kb insert as shown in **Figure 2**. The IRs anneal to form a stem-loop with the DRs at its base.

DNA synthesis was carried out with a fluorescently labeled primer and the reaction products analyzed by agarose gel electrophoresis. Faithful replication of the FXc template generates complete double-stranded parental (P) molecules, which migrate in a retarded position on the gel. A slippage event generates a heteroduplex molecule (H), composed of a parental strand annealed to a recombinant strand lacking one of the DRs and the 1370 bp region between them. Heteroduplex molecules migrate ahead of parental molecules. Stalled (S) replication as the polymerase reaches the base of the hairpin results in a truncated molecule that migrates further than either parental or heteroduplex molecules.

### SLIPPAGE OF *P. abyssi* PolB AND PolD REPLICATIVE POLYMERASES

It has been proposed that PolB and PolD have different roles in the cell, both participating at the replication fork in a manner analogous to *Bacillus subtilis* and the eukaryotic replisome. The current model for *P. abyssi* DNA replication proposes that *Pab*PolD performs RNA-primed DNA synthesis and is later displaced by *Pab*PolB to carry out processive DNA synthesis, at least on the leading strand (Henneke et al., 2005; Rouillon et al., 2007). Because of the capacity of *Pab*PolD to displace RNA primers in a PCNA-dependent manner, it has been suggested that *Pab*PolD is involved in lagging strand replication. However, definitive confirmation using genetic approaches such as those employed with eukaryotic polymerases has yet to be performed.

**FIGURE 3:**
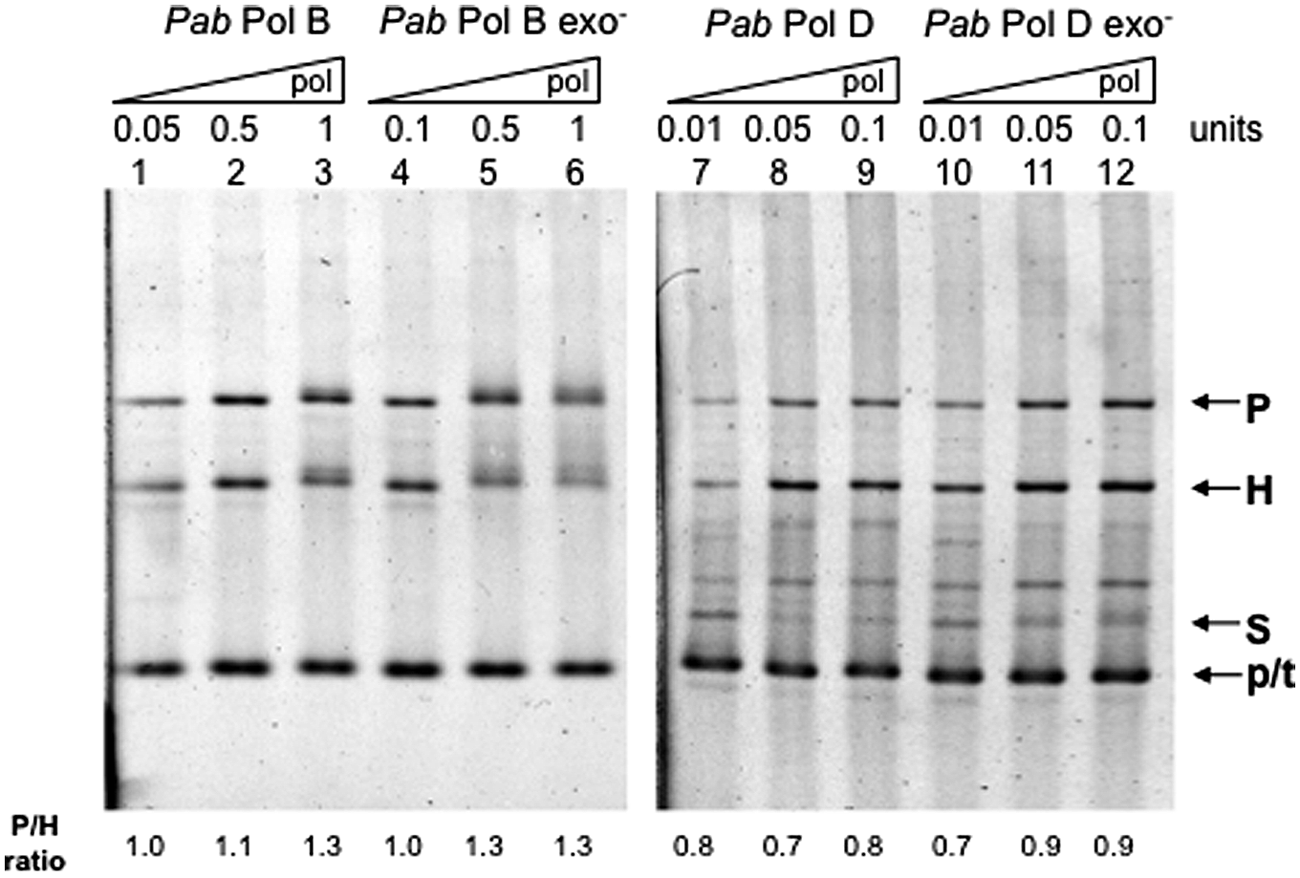
**Effect of DNA polymerase concentration on *Pab* DNA polymerase slippage.** Primer extension reactions were carried out as described in the “Materials and Methods” using increasing amounts of the appropriate *Pab* DNA polymerase. One polymerase unit represents 27 pmol *Pab*PolB: 4 pmol *Pab*PolB exo-; 96 pmol *Pab*PolD; and 104 pmol of *Pab*PolD exo-. P, H, S, and p/t refer to parental, heteroduplex, stalled molecules, and primer-template, respectively. The ratio P/H is indicated below the figure.

In order to test whether their putatively separate roles in leading and lagging strand replication also imply different slippage properties, we examined the slippage efficiency of wildtype *Pab*PolB and *Pab*PolD enzymes using the FXc template (**Figure 2**). *Pab*PolB generated both parental and heteroduplex molecules, which indicate a mixture of normal FXc replication and slippage events (**Figure 3**, lanes 1–3). Similar proportions of parental and heteroduplex molecules were produced by *Pab*PolB, with a slightly higher ratio of parental molecules as the polymerase concentration was increased. *Pab*PolD behaved in a similar way although the proportion of heteroduplex molecules was higher and overall synthesis was improved at higher polymerase concentrations (**Figure 3**, lanes 7–9). These results indicate that *Pab*PolB and *Pab*PolD can slip under our assay conditions. The behavior of *Pab*PolB and *Pab*PolD is similar to that observed for Pol III HE, T7 Pol, or *Taq* Pol that also produce both parental and heteroduplex molecules (Canceill and Ehrlich, 1996; Canceill et al., 1999; Viguera et al., 2001b).

To generate parental molecules, a DNA polymerase must open the hairpin formed by the annealed inverted repeats of the single-stranded template (**Figure 2**), which is largely dependent on a DNA polymerase’s strand displacement activity. As a consequence, polymerases with high strand displacement activity (e.g., ϕ29 DNA polymerase) do not slip while DNA polymerases devoid of strand displacement activity (e.g., *E. coli* Pol II or T4 DNA pol) generate heteroduplex molecules as the sole product of the reaction (Canceill et al., 1999).

Strand displacement activity is modified in some DNA polymerase exo- mutants. For example the T7 DNA polymerase has relatively low strand displacement activity (Canceill et al., 1999). However, the T7 pol exo- (Sequenase^TM^), carrying a 28 amino acid deletion that inactivates its proofreading activity (Engler et al., 1983; Lechner et al., 1983), has increased strand displacement activity that prevents T7 pol exo- slippage (Canceill et al., 1999). Similarly, the *E. coli* Pol II exo- mutant gains a degree of strand displacement activity and the ability to synthesize parental molecules (Canceill et al., 1999). However, not all exo- forms exhibit increased strand displacement activity. For example, an exo- form of F 29 caused by a point mutation shows a 90% reduction in strand displacement activity compared to the native enzyme (Soengas et al., 1992).

To test whether *Pab* pol exo- variants have modified slippage properties, we performed FXc template primer extension assays using exo- mutant forms of *Pab*PolB and *Pab*PolD carrying single point mutations (D215A and H451A, respectively; see Palud et al., 2008). Our results show that the exo- forms of *Pab*PolB and *Pab*PolD both generated heteroduplex and parental molecules. However, unlike their native forms, increasing polymerase concentration inhibited slippage and resulted in a higher proportion of parental molecules (**Figure 3**, lanes 4–6 and 10–12, respectively). Both the native *Pab*PolD enzyme and its exo- form produced some molecules that migrate between the heteroduplex and stalled molecules, possibly the result of inefficient polymerase progression through the hairpin (Viguera et al., 2001a).

### MAGNESIUM CONCENTRATION AFFECTS THE SLIPPAGE OF *P. abyssi* POLYMERASES

The concentration of divalent cations needs to be precisely controlled during DNA synthesis as it affects enzyme activity, enzyme fidelity, primer/template annealing, and the stability of secondary structures, such as the stem-loop used in our assay. The fidelity of *Taq* and *Pfu* DNA polymerases in terms of base substitution and frameshift errors is dependent on magnesium concentration (Eckert and Kunkel, 1990; Cline et al., 1996). Moreover, trinucleotide repeat expansions are produced *in vitro* by* Taq*, *E. coli* Pol I, and the Pol I Klenow fragment at certain magnesium concentrations (Lyons-Darden and Topal, 1999). With respect to slippage, magnesium concentration differentially affects the slippage errors produced by thermostable DNA polymerases (Viguera et al., 2001b). *In vitro* experiments showed that slippage error-derived heteroduplex molecules account for almost all the product generated by *Pfu* DNA polymerase over the magnesium concentration range that permits efficient DNA synthesis (0.5–7.5 mM MgSO_4_). In contrast, from the same template *Taq* DNA polymerase faithfully generates parental molecules at a low magnesium concentration (0.5 mM MgCl_2_), heteroduplex molecules at high magnesium concentrations (10–20 mM MgCl_2_) and a mixture of parental and heteroduplex molecules at intermediate magnesium concentrations (1–7.5 mM MgCl_2_; Viguera et al., 2001b).

**FIGURE 4:**
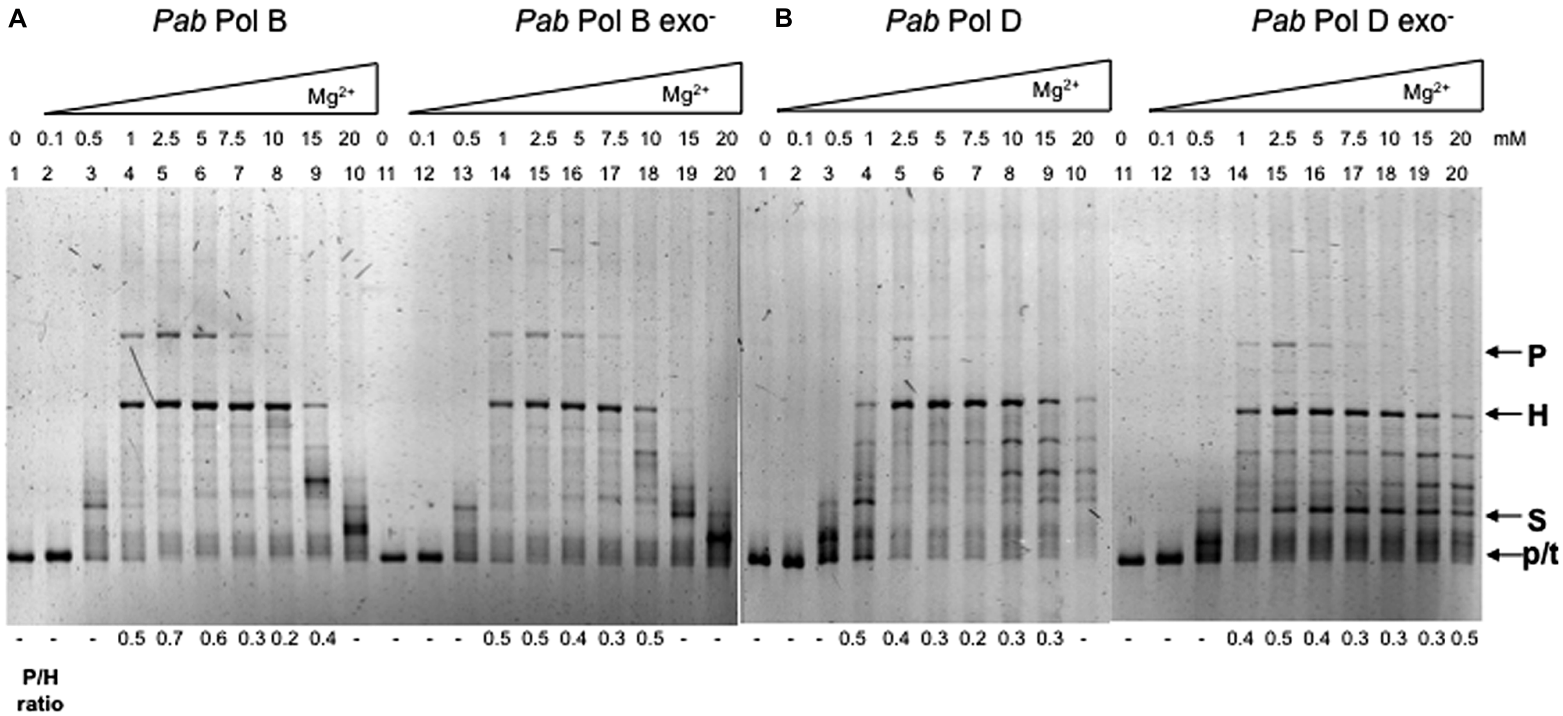
**Effect of magnesium concentration on *Pab* DNA polymerase slippage.** Primer extension reactions were carried out as described in the “Materials and Methods.” Reactions contained 0.5 units of *Pab*PolB, (1.35 pmol), *Pab*PolB exo- (2 pmol), *Pab*PolD (4.8 pmol), and *Pab*PolD exo- (5.2 pmol) with increasing concentrations of MgCI_2_. P, H, S, and p/t refer to parental, heteroduplex, stalled molecules, and primer-template, respectively.

These observations prompted us to analyze the effect of magnesium concentration on the slippage errors produced by the wildtype and exo- forms of *Pab*PolB and *Pab*PolD. We found that varying magnesium concentration affected both slippage and overall DNA synthesis (**Figure 4**). There was almost no synthesis by *Pab*PolB, *Pab*PolD or their exo- forms at low magnesium concentrations (0.1–0.5 mM; **Figure 4A**, lanes 2–3 and 12–13; **Figure 4B**, lanes 2–3 and 12–13). Synthesis was also inhibited at the highest concentrations tested (15–20 mM; **Figure 4A**, lanes 9–10 and 19–20; **Figure 4B**, lanes 9–10 and 19–20). Parental molecules were readily detectable together with heteroduplex molecules at low to medium magnesium concentrations (1–5 mM; **Figure 4A**, lanes 4–6). Increasing magnesium concentration up to 15 mM decreased the proportion of parental molecules and resulted in heteroduplex molecules as the main reaction product (**Figure 4A**, lanes 7–9). This latter result could be due to stabilization of the hairpin structure by high magnesium concentrations making polymerase progression more difficult inside the hairpin (Canceill and Ehrlich, 1996). The effect of magnesium concentration on *Pab*PolB was similar at the 40 and 100 μM nucleotide concentrations tested (data not shown). *Pab*PolB exo- behaved in a similar way to the wildtype enzyme (**Figure 4A**, lanes 14–18).

Although both *Pab*PolD and *Pab*PolD exo- generated parental molecules, the main reaction products were heteroduplex molecules whenever synthesis was efficient (**Figure 4B**, lanes 4–10 and lanes 14–20). Additionally, some of the molecules generated by *Pab*PolD and *Pab*PolD exo- migrated between heteroduplex and stalled molecules (**Figure 4B**, lanes 8–9 and 15–20) that probably represent partially replicated DNA molecules due to inefficient polymerase progression within the hairpin.

In order to confirm that the magnesium concentrations used in the previous experiment are compatible with efficient *Pab*PolB and *Pab*PolD DNA synthesis, we performed primer extension experiments using a 5′ fluorescently labelled primer (33 mer) and a short single-stranded linear DNA template (87 mer) that has the potential to form a 28 bp secondary structure but lacks DRs (Henneke, 2012). This assay should only assess the replication efficiency of a template with a small hairpin without the possibility of slippage between DRs at its base. Fully replicated molecules were generated for both *Pab*PolB and *Pab*PolD using the same range of magnesium concentrations used for the primer extension assays (**Figure S1**), which indicates that the reaction conditions used were optimal for DNA synthesis.

We conclude that in spite of their high fidelity in terms of base substitution, *Pab*PolB and *Pab*PolD are highly prone to slip on ssDNA templates upon encountering secondary structures flanked by DRs, generating parental and heteroduplex molecules in a magnesium concentration-dependent manner. This is in agreement with previous results describing similar behavior for *Taq* DNA polymerase (Viguera et al., 2001b); **Figure S2**. In contrast, native *Pfu* DNA polymerase generates mostly heteroduplex molecules regardless of magnesium concentration (Viguera et al., 2001b); **Figure S2**

### *Pyrococcus abyssi* PCNA CAN MODULATE THE SLIPPAGE OF *Pab*PolB AND *Pab*PolD

The sliding clamp of Archaea, Eukarya, and Bacteria forms a ring around dsDNA that prevents the dissociation of DNA polymerases from their template, thus enhancing processivity (O’Donnell et al., 2013). Moreover, it acts as a platform that regulates polymerase switching, coupling DNA replication and DNA repair (López de Saro, 2009). The *E. coli* sliding clamp β homodimer subunit requires the clamp loader (or γ complex) to load it onto the template. The addition of β to primer extension reactions on a hairpin-containing template favors Pol III HE slippage as the synthesis of heteroduplex molecules is stimulated (Canceill and Ehrlich, 1996).

*Pyrococcus abyssi*, possesses a single processivity clamp, PCNA, that forms a homotrimer (Castrec et al., 2009). In contrast to Bacteria and Eukarya, the archaeal PCNA can be loaded onto DNA without a clamp-loader. *Pab*RF-C and *Pab*PolB, but not *Pab*PolD, enhance PCNA loading.* Pab*RF-C and *Pab*PolB associate with *Pab*PCNA, forming a stable complex on primed DNA (Rouillon et al., 2007).

**FIGURE 5:**
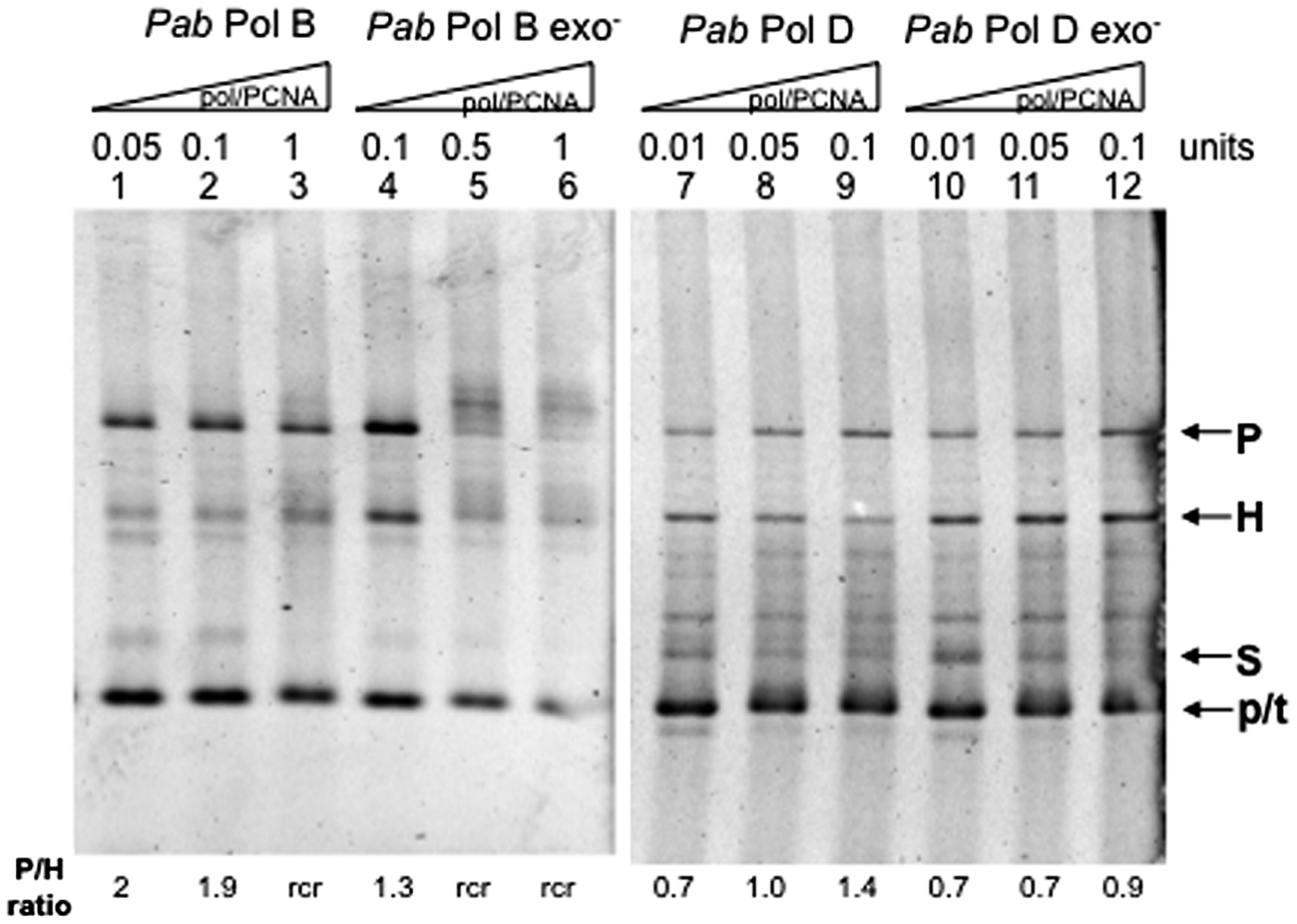
**Effect of PabPCNA on *Pab* DNA polymerase slippage.** Primer extension reactions were carried out as described in the “Materials and Methods” on 25 ng (12.2 fmol)of FXc template using increasing amounts of *Pab* DNA polymerase and equimolar amounts of PabPCNA. One polymerase unit represents 27 pmol *Pab*PolB: 4 pmol *Pab*PolB exo-: 96 pmol *Pab*PolD and 104 pmol of *Pab*PolD exo-, P, H, S, and p/t refer to parental heteroduplex, stalled molecules, and primer-template, respectively. The ratio P/H Is Indicated below the figure. Rolling circle replication (rcr) is indicative of slippage inhibition because of the strand displacement activity of the polymerase.

We therefore investigated the role of PCNA on *Pab*PolB and *Pab*PolD slippage (**Figure 5**). The addition of equimolar amounts of *Pab*PCNA to the FXc replication assay reduced *Pab*PolB slippage. Formation of parental molecules was stimulated and the proportion of heteroduplex molecules diminished (**Figure 5**, lanes 1–3) with respect to reactions performed in the absence of PCNA (compare with **Figure 3**, lanes 1–3). *Pab*PCNA addition also reduced *Pab*PolB exo- slippage (**Figure 5**, lanes 4–6, compare with **Figure 3**, lanes 4–6). Furthermore, the addition of PCNA resulted in the appearance of slowly migrating high molecular weight molecules (**Figure 5**, lanes 5–6). These molecules could be the result of rolling circle replication (*rcr*), which implies that after completion of one round of replication, the newly synthesized strand becomes displaced allowing synthesis to continue (Canceill et al., 1999).

The presence of *Pab*PCNA also increases the proportion of parental versus heteroduplex molecules generated by *Pab*PolD (**Figure 5**, lanes 7–9), indicating that it also represses slippage by this DNA polymerase. However, *Pab*PCNA had only a slight effect on *Pab*PolD exo- slippage (**Figure 5**, lanes 10–12), as the proportion of parental molecules was only slightly higher.

We conclude from these experiments that *Pab*PCNA stimulates the ability of *Pab*PolB and *Pab*PolD to replicate through a hairpin structure by inhibiting slippage, with the strongest effect in terms of slippage inhibition observed on *Pab*PolD exo-. These results agree with those obtained by Henneke et al. (2005) in which *Pab*PCNA stimulated the strand displacement activity of *Pab*PolB and *Pab*PolD (Henneke, 2012).

### THE *Thermus thermophilus* SINGLE-STRANDED DNA BINDING (SSB) PROTEIN DOES NOT AFFECT SLIPPAGE ERRORS PRODUCED BY *Pab* DNA POLS

The amount of slippage exhibited by different DNA polymerases has been shown to be modulated by SSB proteins (Canceill and Ehrlich, 1996; Canceill et al., 1999). *E. coli* SSB stimulates the slippage of Pol III HE, inhibits the slippage of *E. coli* polymerase I and T7 DNA polymerase, and has no effect on *E. coli* pol II or T4 DNA polymerase. On the other hand, T4 SSB protein (gp32) inhibits T4 DNA pol slippage but does not affect the slippage properties of the Pol I Klenow fragment. These contrasting effects of SSB proteins on the same DNA template cannot be understood solely in terms of interaction with DNA, but rather as an interaction between SSB proteins and the different polymerases that alters their strand displacement activity (Canceill et al., 1999).

**FIGURE 6:**
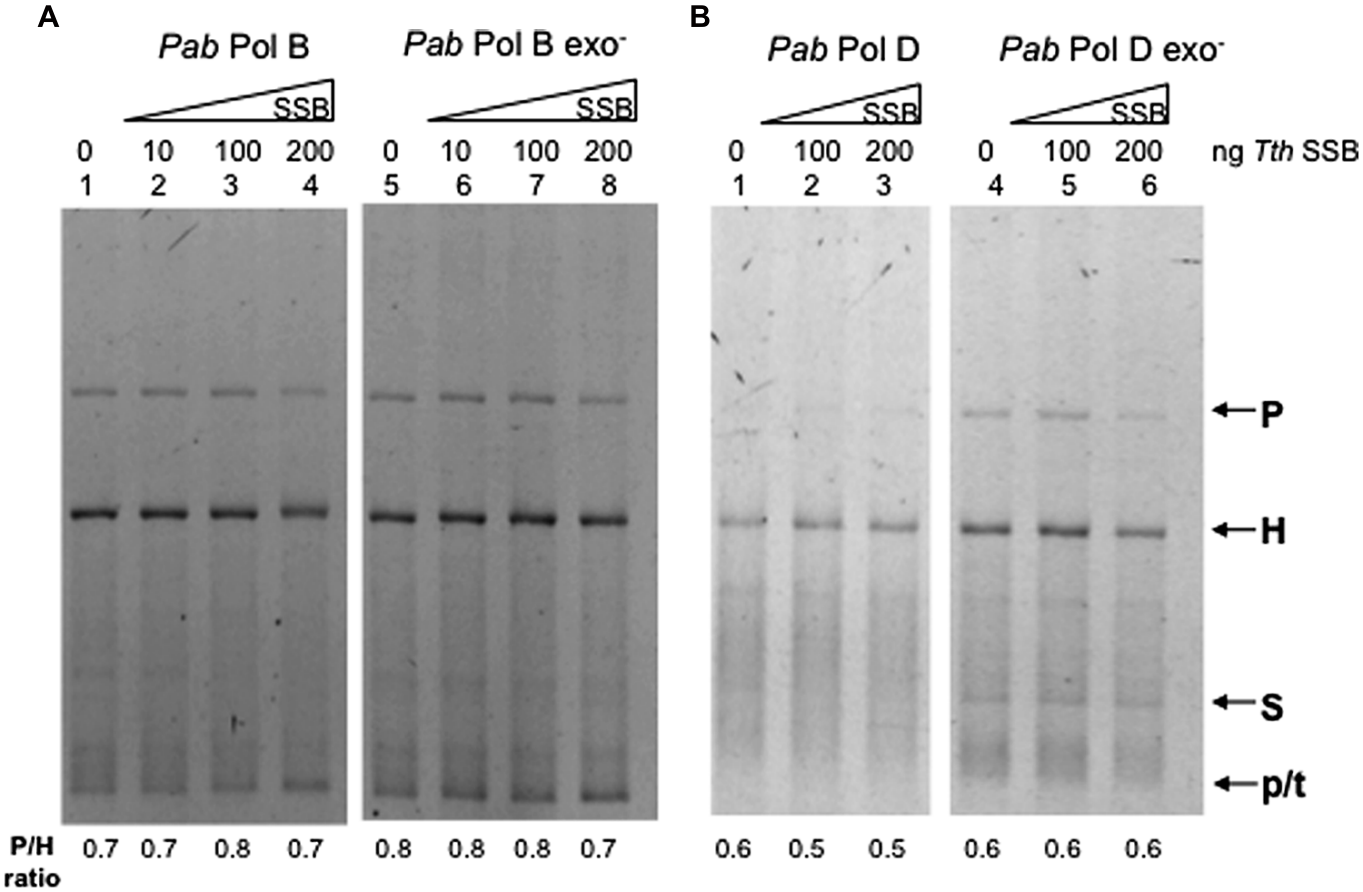
**Effect of *Thermus thermophilus* SSB (*T. thermophilus* SSB) on *Pab* DNA polymerase slippage.** Primer extension reactions were carried out as described in the “Materials and Methods” on 25 ng (12.2 fmol) of FXc template. Reactions contained 1 unit of *Pab*Pol B and *Pab*Pol B exo- (27 and 4 pmol, respectively, **A**), 0.05 units of *Pab*PolD and *Pab*PolD exo- (4.3 and 5.2 pmol respectively, **B**), and were pre-incubated at 60°C with increasing concentrations of *T. thermophilus* SSB (1 ng of *Tth* SSB corresponds to 16.78 fmol). P, H, S, and p/t refer to parental heteroduplex. stalled molecules and primer-template, respectively.

We therefore investigated whether a SSB protein could modify the slippage properties of *Pab* pols. *Thermus thermophilus* (*Tth*) SSB stimulates DNA synthesis of *Tth* DNA polymerase and the heterologous DNA polymerase from the Archaea *P. furiosus* (Perales et al., 2003). Furthermore, *Tth*SSB increases the fidelity of proofreading deficient *Thermus thermophilus* DNA polymerase (Perales et al., 2003). We tested the slippage properties of *Pab*PolB, *Pab*PolD, and their exo- forms in the presence of increasing amounts of *Tth*SSB. Results obtained for *Pab*PolB and its exo- form are shown in **Figure 6A**, lanes 1–8. Heteroduplex products were detected in both the presence and absence of *Tth*SSB. Similar results were obtained for *Pab*PolD and its exo- form (**Figure 6B**, lanes 1–6). We conclude that *Tth*SSB neither stimulated overall synthesis efficiency nor the slippage of *Pab* polymerases under the reaction conditions assayed.

## DISCUSSION

Interest in DNA repeat instability has increased dramatically since links were established between expansions of trinucleotide repeats and neurodegenerative diseases (Bacolla and Wells, 2009; reviewed in Kim and Mirkin, 2013), microsatellite instability and certain types of cancer (Shah et al., 2010) and the identification of frameshift-mediated regulation of gene expression at simple sequence contingency loci of pathogenic bacteria such as *Neisseria* or *Haemophilus* (Moxon et al., 1994; Bayliss et al., 2001; reviewed in Gemayel et al., 2010).

Because of the association between DNA repeat instability and DNA replication, DNA polymerases have been analyzed *in vitro* to establish their ability to replicate repeated DNA sequences. We have shown previously that the main replicative DNA polymerase of the model bacteria *E. coli*, DNA Pol III HE, is able to slip *in vitro* on hairpin-containing templates despite the high fidelity required for genome replication (Canceill et al., 1999). Moreover, slippage is stimulated by factors that affect Pol III processivity such as the presence of β-clamp or SSB proteins (Canceill and Ehrlich, 1996).

Thermostable DNA polymerases are widely used for a number of applications, mostly involving PCR amplification. We have previously shown that replication slippage occurs efficiently even during the first PCR amplification cycle of *Taq* Pol, *Pfu* Pol, Pyra^TM^ Pol (*Pab* PolB exo-), or the Expand^TM^ mixture (*Taq* Pol and *Pwo* Pol; Viguera et al., 2001b). However, no slippage was detected during PCR performed by* Tfu* Pol or Vent^®;^ Pol. Since, high fidelity DNA polymerases can undergo slippage in terms of base substitution, slippage is only inhibited in those polymerases endowed with high strand displacement activity. Thus, the use of DNA polymerases with high strand displacement activity is advisable when amplifying DNA templates with potential strong secondary structures.

In this study, we have shown that replicative *P. abyssi* DNA polymerases are able to slip *in vitro* on hairpin containing templates as heteroduplex molecules, indicative of slippage error, as well as parental molecules were obtained at every enzyme concentration assayed. This result is quite different to those obtained for the thermostable polymerase B from *P. furiosus* (*Pfu*) or the mesophilic DNA polymerases *E. coli* Pol II and T4, where heteroduplex molecules are the only reaction product (Viguera et al., 2001b). Although native *Pfu* Pol generated some parental molecules, the main reaction products were heteroduplex structures (**Figure S2B**). In contrast,* Taq* DNA pol, Pol III HE, Pol I, and T7 DNA pol generate heteroduplex and/or parental molecules depending on the polymerase concentration used in the assay (Canceill et al., 1999; Viguera et al., 2001b; **Figure S2A**). High polymerase concentrations are believed to promote step-by-step progression inside the hairpin via multiple association/dissociation events (Canceill et al., 1999), thus slippage assay sensitivity to polymerase concentration is consistent with a polymerase possessing some degree of strand displacement activity.

Our interpretation is that the different slippage properties of the closely related *Pab*PolB and *Pfu* Pol are most likely due to *Pab*PolB having higher strand displacement activity, which allows it to generate a higher proportion of parental molecules.

Both *Pab*PolB and *Pab*PolD generated heteroduplex molecules alone or a combination of parental and heteroduplex products at the different magnesium concentration tested whenever synthesis was efficient. This result was somewhat similar to that obtained for *Taq* DNA pol, where either parental or heteroduplex molecules were obtained depending on the magnesium concentration (Viguera et al., 2001b), although we did not find any reaction condition where parental molecules were the sole product of either of the *Pab* polymerases. The strand displacement activity of *Pab*PolB and *Pab*PolD is probably insufficient to reliably open and progress through the hairpin structure even at the lowest magnesium concentration tested; a condition that should reduce DNA duplex stability. Consequently, even if *Pab*PolB and *Pab*PolD have different roles at the replication fork, they do not differ in terms of their slippage properties. This result prompted us to study other cellular factors that could inhibit the slippage errors detected by our *in vitro* assay.

We have shown that the *Pab*PCNA sliding clamp promotes the synthesis of parental molecules by *Pab*PolB. This effect is even more prominent for the exonuclease-deficient *Pab*PolB (**Figure 5**). In comparison to *Pab*PolB, inhibition of slippage by PCNA was weaker for *Pab*PolD (**Figure 5**). *Pab*PolB has been identified as the leading strand DNA polymerase (Henneke et al., 2005; Rouillon et al., 2007). *Pab*PCNA interacts with *Pab*PolB in a DNA-dependent way and stimulates its processivity, clamping *Pab*PolB to DNA (Henneke et al., 2005; Henneke, 2012). The higher processivity of the *Pab*PCNA-PolB complex and stimulation of strand displacement activity (Henneke et al., 2005) would facilitate opening of the DNA duplex leading to the synthesis of parental molecules. The effect of *Pab*PCNA is further increased for *Pab*PolB exo-. One possibility is that strand displacement activity is increased to some degree in this mutant and that this facilitates parental formation as has been observed for T7 pol exo- (Sequenase^TM^) and Pol II exo- (Canceill et al., 1999).

In our opinion, this result confirms *Pab*PCNA-PolB as a competent and stable complex, capable of continuously synthesizing the leading strand. Upon encountering secondary structures (such as hairpin loops), DNA synthesis is unperturbed and the *Pab*PCNA-PolB complex is capable of continuing strand elongation.

The reason that *Pab*PCNA inhibited *Pab*PolD replication slippage to a lesser extent than *Pab*PolB is probably due to insufficient stimulation of strand displacement activity (Henneke et al., 2005) under the conditions tested. *Pab*PCNA binds PolB and PolD in different ways (Castrec et al., 2009). Two PCNA-interacting protein (PIP) boxes are needed for *Pab*PolD binding to *Pab*PCNA whereas only one PIP motif is essential for *Pab*PolB binding. This suggests that the mechanism involved in the *Pab*PCNA-mediated stimulation of *Pab*PolD may be different from that involved in *Pab*PolB.

Previous work (Canceill and Ehrlich, 1996) has shown that in *E. coli*, the β-clamp does not stimulate the generation of parental molecules by Pol III HE but instead increases the formation of heteroduplex ones. Our finding that the addition of *Pab*PCNA promotes parental formation by *Pab*PolB and *Pab*PolD, indicates that interaction between DNA polymerases and PCNA in the archaeal *P. abyssi* promotes faithful replication of DNA secondary structures. The functional homology between archaeal and eukaryal proteins, i.e., human PCNA can be loaded onto DNA by the *P. abyssi* RF-C complex (Henneke et al., 2002), suggests that slippage by replicative eukaryal DNA polymerases may also be inhibited by presence of the sliding clamp.

## Conflict of Interest Statement

The Review Editor Bernard Connolly declares that, despite having collaborated and published with Ghislaine Henneke, the review process was handled objectively. All authors declare that the research was conducted in the absence of any commercial or financial relationships that could be construed as a potential conflict of interest.

## SUPPLEMENTARY MATERIAL

The Supplementary Material for this article can be found online at: http://www.frontiersin.org/journal/10.3389/fmicb.2014.00403/abstract
