# Supplementary figures and images for: Replication slippage of the thermophilic DNA polymerases B and D from the Euryarchaeota Pyrococcus abyssi
Source: Front Microbiol. 2014 Aug 7;5:403. doi: 10.3389/fmicb.2014.00403 (PMC4134008; doi:10.3389/fmicb.2014.00403)

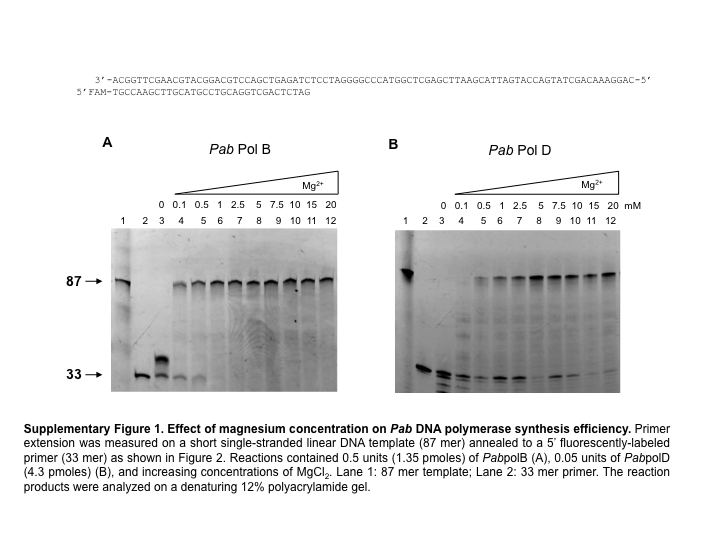

Supplement: Supplementary file 1 [file Image_1.TIF]

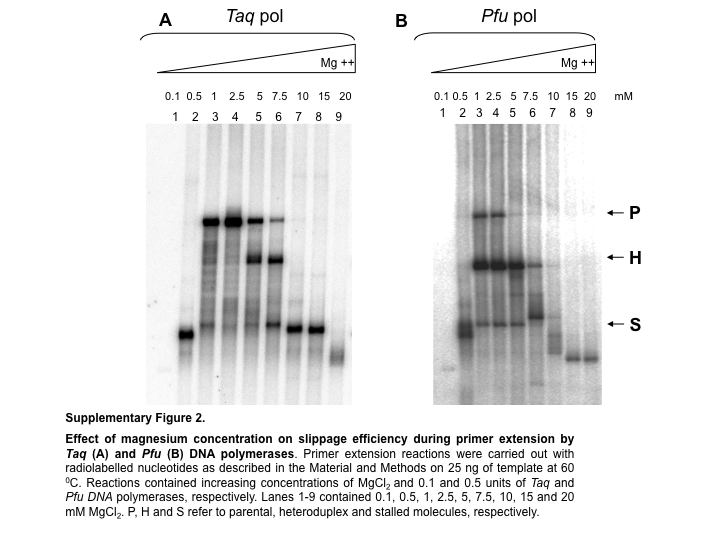

Supplement: Supplementary file 2 [file Image_2.TIF]
